# Outcomes of simultaneous resection for elderly patients with colorectal liver metastasis: A propensity score matching analysis

**DOI:** 10.1002/cam4.4826

**Published:** 2022-05-24

**Authors:** Qichen Chen, Yizhou Zhang, Yiqiao Deng, Zhen Huang, Hong Zhao, Jianqiang Cai

**Affiliations:** ^1^ Department of Hepatobiliary Surgery, National Cancer Center/National Clinical Research Center for Cancer/Cancer Hospital Chinese Academy of Medical Sciences and Peking Union Medical College Beijing China

**Keywords:** colorectal liver metastasis, elderly, propensity score matching, simultaneous resection

## Abstract

**Background:**

Evidence on simultaneous resection for elderly patients (age ≥ 70 years) with colorectal liver metastasis (CRLM) is lacking.

**Methods:**

Four hundred and eighty‐two CRLM patients treated by simultaneous resection were categorised into young group (age < 70 years) and elderly group (age ≥ 70 years). Propensity score matching (PSM1) was performed to adjust for differences in baseline characteristics and compare short‐term outcomes. An additional propensity score matching (PSM2) including short‐term outcomes was performed to analyse survival. Subgroup analysis was performed in patients stratified by the Clinical Risk Score (CRS).

**Results:**

After PSM1, 87 young group patients were matched to 50 elderly group patients. Patients in the elderly group had a significantly higher rate of overall post‐operative complications (68.0% vs. 46.0%, *p* = 0.013). After PSM2, 89 young group patients were matched to 47 elderly group patients. Progression‐free survival (PFS) was comparable between the two groups (median 11.0 months vs. 9.8 months, *p* = 0.346). Age ≥ 70 independently predicted worse overall survival (OS) (Hazard ratio, HR = 2.57, 95% confidence interval, CI 1.37–4.82) in multivariate analysis. In the subgroup multivariate analysis of patients with CRS score 3–5, age ≥ 70 was independently associated with worse PFS (HR = 1.62, 95% CI 1.01–2.62) and OS (HR = 2.34, 95% CI 1.26–4.35).

**Conclusions:**

Simultaneous resection for elderly CRLM patients is acceptable. Further studies are required to determine the optimal treatment for elderly CRLM patients with high CRS scores.

## Graphical abstract

482 CRLM patients treated by simultaneous resection were categorized into the young group (age 70 years) and the elderly group (age GreaterEqual;70 years). Although elderly patients receiving simultaneous resection of CRLM have worse OS, similar PFS was reached in the multivariate analysis after propensity score matching. Simultaneous resection for elderly CRLM patients is acceptable.

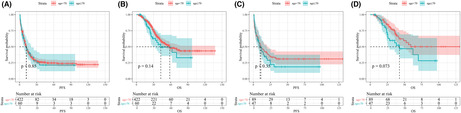

## 1 INTRODUCTION

Surgical resection is currently the treatment of choice for colorectal liver metastasis (CRLM).^1^ Five‐year survival of approximately 50% has been reported for CRLM patients undergoing complete surgical resection.^2^ As the life expectancy of the global population increases, the number of elderly patients with CRLM is also expected to increase.^3^, ^4^ Nonetheless, a significantly lower proportion of elderly patients are eligible for curative surgery for CRLM, and the surgical approaches offered to these patients are also considered to be less aggressive.^5^, ^6^ With the development of surgical techniques and effective chemotherapy regimens, the proportion of elderly CRLM patients receiving surgical resection has been increasing over the years.^5^, ^7^, ^8^ Although many studies have discussed the risks and benefits of surgery for elderly patients with CRLM, the various cut‐off values used to define elderly patients and the contradictory results of these studies have resulted in controversy regarding this issue.^5^, ^9^, ^10^, ^11^, ^12^, ^13^, ^14^, ^15^, ^16^

Approximately 20% of CRLM patients are estimated to have a synchronous presentation, which is defined as liver metastasis discovered at diagnosis or during surgery for the primary tumour.^17^, ^18^ Surgeries for synchronous CRLM can be performed in various sequences, including liver‐first, primary‐first or simultaneous resection.^19^ Compared with staged approaches, simultaneous resection of CRLM leads to similar oncological outcomes in selected patients and is also associated with reduced costs and lengths of stay.^20^, ^21^ However, little is known about the safety and outcomes of elderly patients receiving simultaneous resection of CRLM. In 2004, Tanaka et al. retrospectively reviewed 39 synchronous CRLM patients treated by simultaneous resection and reported that age ≥ 70 years was associated with worse overall survival (OS) on univariate analysis but not on multivariate analysis.^22^ Based on these observations, Tanaka et al. suggested that simultaneous resection should be avoided in elderly patients.^22^ The management of CRLM has changed markedly after 2004 as surgical techniques developed and effective chemotherapy regimens were introduced. In addition, due to the above authors' relatively small study population and failure to demonstrate the prognostic impact of advanced age in the multivariate analysis, whether simultaneous resection should be avoided in elderly patients with synchronous CRLM remains largely unclear.

The aims of this study were to compare the outcomes of elderly (age ≥ 70 years) and young (age < 70 years) synchronous CRLM patients who received simultaneous resection and answer whether simultaneous resection for elderly patients should be avoided.

## 2 METHODS

### 2.1 Study population

This study enrolled 482 consecutive patients who received simultaneous resection of CRLM at Cancer Hospital, Chinese Academy of Medical Sciences between December 2008 and May 2019. The inclusion criteria were as follows: (1) histologically confirmed liver metastasis of colorectal adenocarcinoma and (2) hepatic resection with simultaneous primary colorectal resection. The exclusion criteria were as follows: (1) palliative resection surgery (R2 resection); (2) other malignancies; and (3) loss to follow‐up or incomplete clinical data. To increase the comparability of this study to most relevant articles, 70 years old was selected as the cut‐off to define elderly patients in the present study.^9^, ^11^, ^12^, ^13^, ^14^ Patients were categorised into two groups: the young group (age < 70 years) and the elderly group (age ≥ 70 years). Chronic medical diseases (diabetes, hypertension, cardiac disease, etc.) were defined as comorbidities. The study was approved by the Institutional Review Board of the Cancer Hospital, Chinese Academy of Medical Sciences (ID:NCC2019C‐016) and conformed to the Declaration of Helsinki.

### 2.2 Treatment

As described previously, for CRLM patients with initially unresectable liver metastases or risk factors for recurrence (four or more hepatic lesions; diameter of largest hepatic lesion ≥5 cm; carcinoembryonic antigen [CEA] level ≥ 200 ng/ml; and primary tumour invasion of mesenteric lymph nodes, surrounding tissue or organs discovered by imaging), neoadjuvant chemotherapy was recommended.^23^ After neoadjuvant chemotherapy treatment, patients usually received simultaneous primary colorectal resection with liver resection within 4–6 weeks. Liver resection was defined as major or minor resection, the former of which referred to resection of more than two liver segments.^24^ Generally, simultaneous resection is the preferred approach for synchronous CRLM at this institution. Simultaneous resection is not performed in an emergency setting for symptomatic primary tumour. Rectal primary and/or major hepatectomy is not considered as a contraindication for simultaneous resection. The Child–Pugh score was used to measure preoperative liver function. All patients reached Child–Pugh A prior to receiving simultaneous resection. In the simultaneous surgery, hepatectomy is performed first for the majority of patients (liver‐first simultaneous resection), while primary resection and anastomosis were completed first, followed by hepatectomy for other patients, which was decided at the discretion of the clinicians. Clinical risk scoring system (CRS) scores were calculated as follows: a maximum CRLM diameter greater than 5 cm (1 point); primary lymph node positivity (1 point); CEA > 200.0 ng/ml (1 point); more than 1 CRLM (1 point); and an interval less than 12 months between primary colorectal cancer diagnosis and liver metastasis (1 point).^1^ The final decisions for chemotherapy and surgery were made as a consensus of a multidisciplinary team.

### 2.3 Outcomes

Outcomes were divided into short‐term outcomes (operation time, blood loss, blood transfusion, post0operative complications and the duration of post‐operative hospital stay) and long‐term outcomes (progression‐free survival, PFS; overall survival, OS). The severity of postoperative complications was evaluated according to the Clavien–Dindo classification system; major complications were defined as Clavien–Dindo grade III or IV.^25^ For patients who experienced multiple post‐operative complications, the highest grade was used. Post‐operative complications were divided into general complications (pulmonary, cardiac, hepatic and renal complications and catheter sepsis) and surgery‐related complications (gastrointestinal tract leakage, gastrointestinal tract necrosis, intrathoracic or intraabdominal abscess, haemorrhage, ileus, etc.).^26^ Overall survival (OS) was defined as the time between the date of resection and the date of death or the last follow‐up, and progression‐free survival (PFS) was defined as the interval from resection to tumour progression or the last follow‐up.

### 2.4 Statistical analysis

Statistical analyses were performed by R software Version 4.0.2 (http://www.r‐project.org). For the investigated clinicopathologic parameters, categorical variables were compared using the chi‐squared test or Fisher exact test. All continuous variables were analysed using the Mann–Whitney *U* test. Survival curves were estimated with the Kaplan–Meier method and compared by a log‐rank test. All predictors with *p* < 0.10 by univariate analysis were retained in multivariate models. Multivariate analysis using Cox proportional hazards regression analysis was performed to investigate independent factors of survival. Propensity score matching (PSM1) was performed to adjust for differences in baseline characteristics between the two groups and compare short‐term outcomes. As short‐term outcomes were reported to affect patient survival,^27^, ^28^ additional propensity score matching (PSM2) including short‐term outcomes was performed to analyse long‐term outcomes. Propensity score matching was performed with the ‘MatchIt’ package of the R software. Both PSM1 and PSM2 were performed in a 1:2 ratio, by the ‘nearest’ method, with a calliper value of 0.05 and without replacement. Statistical significance was set at a two‐sided *p* < 0.05.

## 3 RESULTS

### 3.1 Patient characteristics

Four hundred eighty‐two patients who underwent simultaneous resection of CRLM were included in this study. The median age of the study population was 59.0 (Interquartile range, IQR 52–65) years. The young group consisted of 422 (87.6%) patients who were younger than 70 years, while the elderly group included 60 (12.4%) patients who were at least 70 years old. Three hundred thirteen (64.9%) patients were female, and 204 (42.3%) patients had comorbidities. Two hundred seventy‐nine (57.9%) patients had primary tumours located in the colon, and 353 (73.2%) patients had primary lymph node metastases. Two hundred seventy‐three (56.6%) patients had multiple liver metastases, and 184 (38.2%) had a bilobular liver metastasis distribution. Forty‐six (9.5%) patients also had extrahepatic metastases. In total, laparoscopic surgeries were performed in 106 (22.0%) patients, while 138 (28.6%) patients received totally open surgery. Forty‐six (9.5%) patients received concomitant radiofrequency ablation (RFA). Two hundred sixty‐seven (55.4%) patients received neoadjuvant chemotherapy, while 313 (64.9%) patients received adjuvant chemotherapy. The detailed patient characteristics are listed in Table 1.

**TABLE 1:** Demographics, clinicopathological characteristic and treatment details before and after propensity score matching

		Before PSM			After PSM1	
	Age < 70 (*n* = 422)	Age ≥ 70 (*n* = 60)	*p*	Age < 70 (*n* = 87)	Age ≥ 70 (*n* = 50)	*p*
Female (%)	267 (63.3)	46 (76.7)	0.042	61 (70.1)	36 (72.0)	0.815
BMI ≥ 24 (%)	194 (46.0)	33 (55.0)	0.19	48 (55.2)	25 (50.0)	0.559
Comorbidity (%)	167 (39.6)	37 (61.7)	0.001	48 (55.2)	30 (60.0)	0.583
ASA score ≥ 3 (%)	40 (9.5)	19 (31.7)	<0.001	17 (19.5)	11 (22.0)	0.731
Primary site in colon (%)	236 (55.9)	43 (71.7)	0.021	59 (67.8)	33 (66.0)	0.828
Primary site in right hemicolon (%)	82 (19.4)	17 (28.3)	0.11	22 (25.3)	15 (30.0)	0.55
Bilobular distribution (%)	164 (38.9)	20 (33.3)	0.409	40 (46.0)	18 (36.0)	0.255
Number of liver metastases ≥2 (%)	244 (57.8)	29 (48.3)	0.165	53 (60.9)	26 (52.0)	0.309
Diameter of largest liver lesion ≥3 cm (%)	181 (42.9)	26 (43.3)	0.948	42 (48.3)	20 (40.0)	0.349
Poor differentiation (%)	134 (31.8)	23 (38.3)	0.309	32 (36.8)	18 (36.0)	0.927
Primary tumour T stage 3 or 4 (%)	390 (92.4)	55 (91.7)	0.838	74 (85.1)	45 (90.0)	0.41
Primary lymph node metastasis (%)	316 (74.9)	37 (61.7)	0.031	58 (66.7)	32 (64.0)	0.752
CEA≥10 ng/μl (%)	190 (45.0)	34 (56.7)	0.091	49 (56.3)	28 (56.0)	0.971
Extrahepatic metastasis (%)	41 (9.7)	5 (8.3)	0.733	10 (11.5)	5 (10.0)	0.787
CRS score 3–5 (%)	212 (50.2)	23 (38.3)	0.084	41 (47.1)	21 (42.0)	0.562
Liver‐first simultaneous resection (%)	286 (67.8)	40 (66.7)	0.864	66 (75.9)	35 (70.0)	0.453
Surgery procedure (%)			0.297			0.785
Totally laparoscopic	90 (21.3)	16 (26.7)		21 (24.1)	11 (22.0)	
Mixed surgery	214 (50.7)	24 (40.0)		43 (49.4)	23 (46.0)	
Totally open surgery	118 (28.0)	20 (33.3)		23 (26.4)	16 (32.0)	
R0 resection (%)	318 (75.4)	46 (76.7)	0.825	67 (77.0)	39 (78.0)	0.894
Concomitant RFA (%)	39 (9.2)	7 (11.7)	0.55	9 (10.3)	5 (10.0)	0.949
Major hepatic resection (%)	204 (48.3)	22 (36.7)	0.09	45 (51.7)	20 (40.0)	0.186
Pringle manoeuvre (%)	288 (68.2)	41 (68.3)	0.989	63 (72.4)	35 (70.0)	0.763
Neoadjuvant chemotherapy (%)	237 (56.2)	30 (50.0)	0.369	51 (58.6)	25 (50.0)	0.328
Adjuvant chemotherapy (%)	276 (65.4)	37 (61.7)	0.57	55 (63.2)	33 (66.0)	0.744

### 3.2 Short‐term outcomes

The median age in the young group was 57 years (IQR 50–62) compared to a median age of 73 (IQR 71–75) in the elderly group (*p* < 0.001). The median post‐operative hospital stay duration was 10 days (IQR 9–13), and the median operative time was 325 min (IQR [260, 415]). The median intraoperative blood loss volume was 200 ml (IQR [100, 400]), and 114 (23.7%) patients received intraoperative blood transfusion. Post‐operative complications were recorded in 234 (48.5%) patients, and 99 (20.5%) patients experienced major complications. Compared to the young group, the elderly group had significantly more comorbidities (61.7% vs. 39.6%, *p* = 0.001), significantly higher proportions of ASA III patients (31.7% vs. 9.5%, *p* < 0.001) and female patients (76.7% vs. 63.3%, *p* = 0.042), and significantly fewer primary lymph node metastases (61.7% vs. 74.9%, *p* = 0.031). Patients in the elderly group had a significantly higher rate of overall post‐operative complications (65% vs. 46.2%, *p* = 0.006). When classified according to no complications, minor complications and major complications based on the Clavien–Dindo scoring system,^25^ patients in the elderly group experienced both more minor complications and more major complications (*p* = 0.024). The post‐operative hospital stay duration, operative time, intraoperative blood loss volume and blood transfusion rate were all similar between the two groups.

To adjust for differences in baseline characteristics between the two groups, we performed 1:2 propensity score matching (PSM1) to evaluate short‐term outcomes. All baseline characteristics in Table 1 except for age, including demographics, clinicopathologic characteristics, and features of surgery or chemotherapy, were included in a generalised linear model as categorical factors to generate the propensity score for PSM1. CRS scores were not included because they were calculated from linear combinations of other variables. One hundred thirty‐seven patients, including 87 in the young group and 50 in the elderly group, were retained in the dataset after PSM1. All baseline characteristics were generally well balanced between the two groups after PSM1. After PSM1, patients in the elderly group had a significantly higher rate of overall post‐operative complications (68.0% vs. 46.0%, *p* = 0.013). When classified according to no complications, minor complications and major complications based on the Clavien–Dindo scoring system,^25^ patients in the elderly group experienced more minor and major complications (*p* = 0.045). The post‐operative hospital stay duration, operative time, intraoperative blood loss volume, blood transfusion rate, general complications and surgery‐related complications were all comparable between the two groups. Details of the short‐term outcomes are listed in Table 2 and Table S1.

**TABLE 2:** Short‐term outcomes before and after propensity score matching

		Before PSM			After PSM1	
	Age < 70 (*n* = 422)	Age ≥ 70 (*n* = 60)	*p*	Age < 70 (*n* = 87)	Age ≥ 70 (*n* = 50)	*p*
Post‐operative hospital stay/d (median [IQR])	10.00 [9.00, 13.00]	11.00 [9.00, 14.00]	0.379	10.00 [9.00, 13.00]	11.50 [9.00, 15.75]	0.226
Blood loss/ml (median [IQR])	200.00 [100.00, 400.00]	200.00 [100.00, 425.00]	0.139	200.00 [175.00, 500.00]	200.00 [100.00, 475.00]	0.081
Operation time/min (median [IQR])	325.00 [260.00, 420.00]	322.50 [265.00, 376.25]	0.276	340.00 [254.00, 434.50]	322.50 [247.50, 402.50]	0.205
Transfusion (%)	98 (23.2)	16 (26.7)	0.557	25 (28.7)	15 (30.0)	0.875
Complication (%)	195 (46.2)	39 (65.0)	0.006	40 (46.0)	34 (68.0)	0.013
Clavien–Dindo grade (%)			0.024			0.045
No complications	227 (53.8)	21 (35.0)		47 (54.0)	16 (32.0)	
Minor (1–2)	112 (26.5)	23 (38.3)		24 (27.6)	20 (40.0)	
Major (3–4)	83 (19.7)	16 (26.7)		16 (18.4)	14 (28.0)	
Surgery‐related complication (%)	124 (29.4)	24 (40.0)	0.095	28 (32.2)	22 (44.0)	0.167
General complications (%)	134 (31.8)	24 (40)	0.203	28 (32.2)	21 (42.0)	0.249

### 3.3 Long‐term outcomes

**FIGURE 1:**
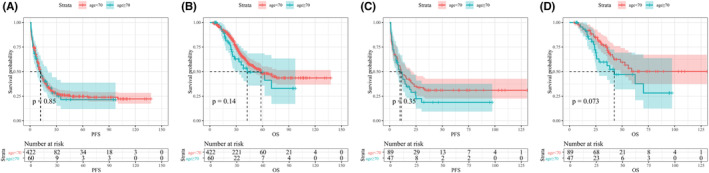
Kaplan–Meier survival plot comparing PFS or OS between the young group and the elderly group. (A) PFS in unmatched full cohort; (B) OS in unmatched full cohort; (C) PFS after PSM2; (D) OS after PSM2

In the full unmatched cohort, the Kaplan–Meier method and log‐rank test revealed no significant differences in PFS (median, young group 11.7 months vs. elderly group 12.0 months, *p* = 0.853) and OS (median, young group 58.3 months vs. elderly group 42.6 months, *p* = 0.142) between the two groups (Figure 1A,B). Short‐term outcomes have been reported to affect the PFS and OS of patients with CRLM.^27^, ^28^ To adjust for differences in baseline characteristics and short‐term outcomes between the two groups, we performed additional 1:2 propensity score matching (PSM2) to evaluate long‐term outcomes. All variables in the generalised linear model for PSM1 plus short‐term outcomes were included in the generalised linear model for PSM2. As shown in Table 3, 136 patients, including 89 in the young group and 47 in the elderly group, were retained in the dataset after PSM2. Baseline characteristics and short‐term outcomes were all similar between the two groups after PSM2. The Kaplan–Meier method and log‐rank test revealed no significant association between age and PFS (median 11.0 months vs. 9.8 months, *p* = 0.346) but showed a trend of the elderly group having worse OS (median, young group not reached vs. elderly group 42.6 months, *p* = 0.073, Figure 1C,D). In the full unmatched cohort, univariate analysis revealed no significant association between age and PFS (*p* = 0.856) or OS (*p* = 0.144, Table 4). In the dataset after PSM2, univariate analysis identified no significant association between age and PFS (*p* = 0.355). However, age ≥ 70 years was identified as a potential risk factor for OS after PSM2 (*p* = 0.077, hazard ratio, HR = 1.67, 95% confidence interval, CI 0.947–2.95). Subsequent multivariate analysis identified age ≥ 70 years (*p* = 0.004, HR = 2.57, 95% CI 1.37–4.82), primary lymph node metastasis (*p* = 0.003, HR = 3.59, 95% CI 1.66–7.75), extrahepatic metastasis (*p* = 0.002, HR = 5.20, 95% CI 1.90–14.2), major hepatic resection (*p* = 0.019, HR = 3.18, 95% CI 1.21–8.32) and general complications (*p* = 0.002, HR = 0.317, 95% CI 0.155–0.646) as independent predictors of OS (Table 5).

**TABLE 3:** Demographics, clinicopathological characteristics, treatment details and short‐term outcomes before and after propensity score matching including short‐term outcomes

		Before PSM			After PSM2	
	Age < 70 (*n* = 422)	Age ≥ 70 (*n* = 60)	*p*	Age < 70 (*n* = 89)	Age ≥ 70 (*n* = 47)	*p*
Female (%)	267 (63.3)	46 (76.7)	0.042	62 (69.7)	35 (74.5)	0.556
BMI ≥ 24 (%)	194 (46.0)	33 (55.0)	0.19	46 (51.7)	24 (51.1)	0.945
Comorbidity (%)	167 (39.6)	37 (61.7)	0.001	43 (48.3)	26 (55.3)	0.437
ASA score ≥ 3 (%)	40 (9.5)	19 (31.7)	<0.001	13 (14.6)	9 (19.1)	0.494
Primary site in colon (%)	236 (55.9)	43 (71.7)	0.021	65 (73.0)	33 (70.2)	0.727
Primary site in right hemicolon (%)	82 (19.4)	17 (28.3)	0.11	28 (31.5)	13 (27.7)	0.646
Bilobular distribution (%)	164 (38.9)	20 (33.3)	0.409	36 (40.4)	17 (36.2)	0.627
Number of liver metastases ≥2 (%)	244 (57.8)	29 (48.3)	0.165	48 (53.9)	26 (55.3)	0.877
Diameter of largest liver lesion ≥ 3 cm (%)	181 (42.9)	26 (43.3)	0.948	42 (47.2)	21 (44.7)	0.78
Poor differentiation (%)	134 (31.8)	23 (38.3)	0.309	33 (37.1)	18 (38.3)	0.889
Primary tumour T stage 3 or 4 (%)	390 (92.4)	55 (91.7)	0.838	79 (88.8)	43 (91.5)	0.619
Primary lymph node metastasis (%)	316 (74.9)	37 (61.7)	0.031	58 (65.2)	31 (66.0)	0.927
CEA ≥ 10 ng/μl (%)	190 (45.0)	34 (56.7)	0.091	52 (58.4)	24 (51.1)	0.411
Extrahepatic metastasis (%)	41 (9.7)	5 (8.3)	0.733	9 (10.1)	4 (8.5)	0.763
CRS score 3–5 (%)	212 (50.2)	23 (38.3)	0.084	38 (42.7)	20 (42.6)	0.987
Liver‐first simultaneous resection (%)	286 (67.8)	40 (66.7)	0.864	57 (64.0)	32 (68.1)	0.638
Surgery procedure (%)			0.297			0.522
Totally laparoscopic	90 (21.3)	16 (26.7)		21 (23.6)	10 (21.3)	
Mixed surgery	214 (50.7)	24 (40.0)		31 (34.8)	21 (44.7)	
Totally open surgery	118 (28.0)	20 (33.3)		37 (41.6)	16 (34.0)	
R0 resection (%)	318 (75.4)	46 (76.7)	0.825	67 (75.3)	37 (78.7)	0.653
Concomitant RFA (%)	39 (9.2)	7 (11.7)	0.55	10 (11.2)	5 (10.6)	0.916
Major hepatic resection (%)	204 (48.3)	22 (36.7)	0.09	38 (42.7)	19 (40.4)	0.799
Pringle manoeuvre (%)	288 (68.2)	41 (68.3)	0.989	63 (70.8)	33 (70.2)	0.944
Neoadjuvant chemotherapy (%)	237 (56.2)	30 (50.0)	0.369	43 (48.3)	21 (44.7)	0.686
Adjuvant chemotherapy (%)	276 (65.4)	37 (61.7)	0.57	60 (67.4)	26 (55.3)	0.164
Post‐operative hospital stay/d (median [IQR])	10.00 [9.00, 13.00]	11.00 [9.00, 14.00]	0.379	11.00 [9.00, 14.00]	11.00 [9.00, 14.00]	0.783
Blood loss/ml (median [IQR])	200.00 [100.00, 400.00]	200.00 [100.00, 425.00]	0.139	200.00 [100.00, 300.00]	200.00 [100.00, 350.00]	0.458
Operation time/min (median [IQR])	325.00 [260.00, 420.00]	322.50 [265.00, 376.25]	0.276	300.00 [227.00, 395.00]	331.00 [276.00, 407.50]	0.414
Transfusion (%)	98 (23.2)	16 (26.7)	0.557	21 (23.6)	11 (23.4)	0.98
Complication (%)	195 (46.2)	39 (65.0)	0.006	53 (59.6)	28 (59.6)	0.998
Clavien–Dindo grade (%)			0.024			0.988
No complication	227 (53.8)	21 (35.0)		36 (40.4)	19 (40.4)	
Minor (1–2)	112 (26.5)	23 (38.3)		35 (39.3)	18 (38.3)	
Major (3–4)	83 (19.7)	16 (26.7)		18 (20.2)	10 (21.3)	
Surgery‐related complication (%)	124 (29.4)	24 (40.0)	0.095	34 (38.2)	16 (34.0)	0.632
General complications (%)	134 (31.8)	24 (40)	0.203	33 (37.1)	19 (40.4)	0.702

**TABLE 4:** Univariate and multivariate analyses of predictive factors for PFS and OS before propensity score matching

	PFS				OS			
	Univariate analysis		Multivariate analysis		Univariate analysis		Multivariate analysis	
Factor	*p*	HR (95% CI)	*p*	HR (95% CI)	*p*	HR (95% CI)	*p*	HR (95% CI)
Age ≥ 70	0.856	1.03 (0.743–1.43)			0.144	1.38 (0.896–2.12)		
Female	0.62	1.06 (0.846–1.32)			0.423	0.883 (0.65–1.2)		
BMI ≥ 24	0.348	1.11 (0.894–1.37)			0.795	1.04 (0.773–1.4)		
Comorbidity	0.901	1.01 (0.816–1.26)			0.869	0.975 (0.721–1.32)		
ASA score ≥ 3	0.635	0.924 (0.666–1.28)			0.432	0.829 (0.52–1.32)		
Primary site in colon	0.631	0.948 (0.764–1.18)			0.975	0.995 (0.737–1.34)		
Primary site in right hemicolon	0.969	0.995 (0.761–1.3)			0.587	1.11 (0.769–1.59)		
Bilobular distribution	<0.001	1.79 (1.45–2.23)	0.999	1 (0.726–1.38)	0.003	1.59 (1.17–2.14)	0.217	0.759 (0.489–1.18)
Number of liver metastases ≥ 2	<0.001	1.73 (1.39–2.17)	0.374	1.15 (0.843–1.58)	<0.001	1.7 (1.25–2.32)	0.471	1.17 (0.763–1.80)
Diameter of largest liver lesion ≥ 3 cm	0.017	1.3 (1.05–1.61)	0.153	1.19 (0.938–1.50)	0.037	1.38 (1.02–1.86)	0.495	1.12 (0.807–1.56)
Poor differentiation	0.035	1.28 (1.02–1.61)	0.350	1.12 (0.883–1.42)	0.121	1.29 (0.934–1.79)		
Primary tumour T stage 3 or 4	0.11	1.43 (0.922–2.23)			0.010	3.25 (1.34–7.92)	0.011	3.26 (1.32–8.07)
Primary lymph node metastasis	<0.001	2.07 (1.58–2.71)	<0.001	1.89 (1.43–2.50)	<0.001	2.71 (1.77–4.14)	<0.001	2.88 (1.86–4.44)
CEA ≥ 10 ng/μl	0.955	1.01 (0.812–1.25)			0.165	1.23 (0.917–1.66)		
Extrahepatic metastasis	<0.001	2.07 (1.48–2.9)	<0.001	1.79 (1.27–2.53)	0.299	1.3 (0.794–2.11)		
Liver‐first simultaneous resection	0.004	1.42 (1.12–1.79)	0.315	1.17 (0.862–1.58)	0.067	1.36 (0.98–1.87)	0.127	1.40 (0.910–2.15)
Surgery procedure								
Totally laparoscopic		Referent		Referent		Referent		Referent
Mixed surgery	0.002	1.61 (1.12–2.16)	0.591	1.10 (0.780–1.55)	0.017	1.79 (1.11–2.88)	0.483	0.821 (0.473–1.42)
Totally open surgery	0.14	1.28 (0.923–1.77)	0.952	1.01 (0.713–1.43)	0.012	1.90 (1.16–3.11)	0.230	1.39 (0.813–2.36)
R0 resection	<0.001	0.547 (0.432–0.694)	<0.001	0.624 (0.482–0.809)	<0.001	0.542 (0.396–0.742)	0.222	0.802 (0.564–1.14)
Concomitant RFA	0.002	1.73 (1.23–2.41)	0.076	1.39 (0.967–1.99)	<0.001	2.09 (1.41–3.11)	0.018	1.69 (1.10–2.61)
Major hepatic resection	<0.001	1.87 (1.5–2.32)	0.014	1.48 (1.08–2.01)	<0.001	1.96 (1.45–2.65)	0.014	1.72 (1.12–2.65)
Pringle manoeuvre	0.027	1.3 (1.03–1.64)	0.027	0.710 (0.525–0.961)	0.019	1.47 (1.07–2.03)	0.292	0.789 (0.509–1.23)
Neoadjuvant chemotherapy	0.298	1.12 (0.904–1.39)			0.009	1.51 (1.11–2.05)	0.299	1.21 (0.848–1.72)
Adjuvant chemotherapy	0.607	0.943 (0.753–1.18)			<0.001	0.567 (0.42–0.767)	<0.001	0.482 (0.350–0.664)
Blood loss ≥ 200 ml	0.052	1.27 (0.999–1.61)	0.925	1.01 (0.778–1.32)	0.152	1.27 (0.915–1.76)		
Operation time ≥ 325 min	<0.001	1.56 (1.26–1.94)	0.044	1.30 (1.01–1.67)	<0.001	1.89 (1.39–2.56)	0.025	1.54 (1.06–2.26)
Transfusion	0.357	1.12 (0.878–1.44)			0.017	1.49 (1.07–2.07)	0.184	1.28 (0.891–1.83)
Post‐operative hospital stay ≥ 10 days	0.222	1.15 (0.921–1.43)			0.007	1.56 (1.13–2.15)	0.144	1.30 (0.913–1.86)
Clavien–Dindo grade								
No complication		Referent		Referent		Referent		Referent
Minor	0.217	1.17 (0.912–1.502)	0.288	1.15 (0.889–1.49)	0.140	1.29 (0.920–1.81)	0.920	1.02 (0.712–1.46)
Major	0.036	1.35 (1.02–1.78)	0.368	1.14 (0.853–1.54)	0.089	1.42 (0.948–2.11)	0.636	0.900 (0.582–1.39)
Surgery‐related complication	0.161	1.18 (0.936–1.49)			0.123	1.29 (0.934–1.77)		
General complications	0.192	1.16 (0.927–1.46)			0.499	1.12 (0.811–1.54)		

**TABLE 5:** Univariate and multivariate analyses of predictive factors for PFS and OS after propensity score matching including short‐term outcomes

	PFS				OS			
	Univariate analysis		Multivariate analysis		Univariate analysis		Multivariate analysis	
Factor	*p*	HR (95% CI)	*p*	HR (95% CI)	*p*	HR (95% CI)	*p*	HR (95% CI)
Age ≥ 70	0.355	1.22 (0.8–1.86)			0.077	1.67 (0.947–2.95)	0.004	2.57 (1.37–4.82)
Female	0.184	1.37 (0.861–2.18)			0.959	0.984 (0.53–1.83)		
BMI≥24	0.521	0.876 (0.584–1.31)			0.855	0.949 (0.545–1.65)		
Comorbidity	0.59	1.12 (0.745–1.68)			0.323	1.33 (0.756–2.34)		
ASA score ≥3	0.924	1.03 (0.591–1.78)			0.454	1.29 (0.66–2.53)		
Primary site in colon	0.703	1.09 (0.692–1.73)			0.839	1.07 (0.567–2.01)		
Primary site in right hemicolon	0.209	0.746 (0.472–1.18)			0.418	0.775 (0.417–1.44)		
Bilobular distribution	<0.001	2.02 (1.34–3.04)	0.590	1.19 (0.637–2.21)	<0.001	2.71 (1.55–4.75)	0.963	1.02 (0.435–2.40)
Number of liver metastases ≥ 2	0.023	1.62 (1.07–2.47)	0.150	0.601 (0.301–1.20)	0.003	2.56 (1.41–4.66)	0.424	0.672 (0.254–1.78)
Diameter of largest liver lesion ≥ 3 cm	0.257	1.26 (0.843–1.9)			0.746	1.1 (0.628–1.91)		
Poor differentiation	0.243	1.28 (0.845–1.94)			0.651	0.869 (0.472–1.6)		
Primary tumour T stage 3 or 4	0.207	1.6 (0.772–3.3)			0.106	3.21 (0.779–13.2)		
Primary lymph node metastasis	<0.001	2.31 (1.45–3.68)	0.003	2.11 (1.29–3.44)	0.005	2.82 (1.37–5.81)	0.003	3.59 (1.66–7.75)
CEA ≥ 10 ng/μl	0.621	1.11 (0.735–1.67)			0.078	1.71 (0.943–3.1)		
Extrahepatic metastasis	0.002	2.7 (1.45–5.04)	0.033	2.06 (1.06–3.98)	0.018	2.68 (1.18–6.05)	0.002	5.20 (1.90–14.2)
Liver‐first simultaneous resection	0.044	1.57 (1.01–2.44)	0.017	1.74 (1.10–2.74)	0.045	1.86 (1.01–3.41)	0.359	1.36 (0.702–2.65)
Surgery procedure								
Totally laparoscopic		Referent				Referent		
Mixed surgery	0.501	1.20 (0.708–2.03)			0.588	1.25 (0.553–2.84)		
Totally open surgery	0.362	0.778 (0.454–1.34)			0.606	1.23 (0.566–2.65)		
R0 resection	<0.001	0.461 (0.293–0.725)	0.016	0.547 (0.334–0.895)	0.007	0.444 (0.246–0.799)	0.104	0.580 (0.301–1.12)
Concomitant RFA	0.052	1.8 (0.995–3.24)	0.319	1.38 (0.733–2.60)	0.023	2.24 (1.12–4.49)	0.050	2.21 (1–4.89)
Major hepatic resection	0.002	1.96 (1.31–2.96)	0.048	1.99 (1.01–3.95)	<0.001	2.84 (1.61–5)	0.019	3.18 (1.21–8.32)
Pringle manoeuvre	0.798	1.06 (0.681–1.65)			0.040	1.94 (1.03–3.65)	0.656	1.19 (0.550–2.59)
Neoadjuvant chemotherapy	0.153	0.743 (0.494–1.12)			0.055	1.73 (0.99–3.03)	0.204	1.63 (0.768–3.46)
Adjuvant chemotherapy	0.304	1.25 (0.816–1.92)			0.769	0.918 (0.518–1.63)		
Blood loss ≥ 200 ml	0.123	1.43 (0.907–2.27)			0.329	1.36 (0.733–2.53)		
Operation time ≥ 325 min	0.224	1.29 (0.857–1.93)			0.004	2.3 (1.3–4.05)	0.294	1.44 (0.729–2.84)
Transfusion	0.205	1.35 (0.849–2.15)			0.080	1.7 (0.94–3.09)	0.678	0.869 (0.449–1.68)
Post‐operative hospital stay ≥ 10 d	0.703	0.921 (0.605–1.4)			0.309	1.39 (0.737–2.63)		
Clavien–Dindo grade								
No complication		Referent				Referent		
Minor	0.837	0.953 (0.604–1.51)			0.180	0.659 (0.359–1.21)		
Major	0.487	1.21 (0.705–2.08)			0.202	0.576 (0.247–1.35)		
Surgery‐related complication	0.901	0.973 (0.637–1.49)			0.392	0.771 (0.425–1.4)		
General complications	0.921	0.979 (0.644–1.49)			0.054	0.542 (0.292–1.01)	0.002	0.317 (0.155–0.646)

### 3.4 Subgroup analysis

**FIGURE 2:**
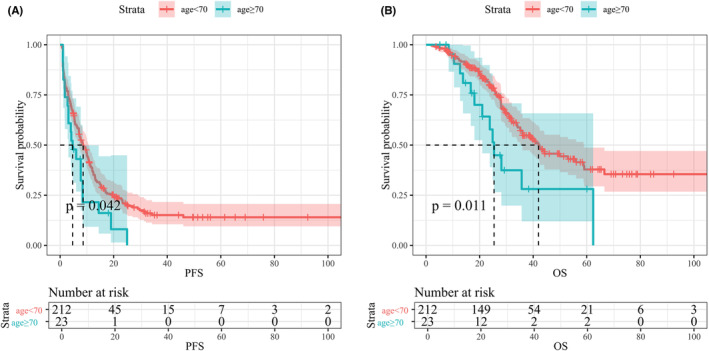
Survival plot comparing survival in subgroup analyses of patients with CRS score 3–5. (A) PFS (B) OS

Given that the CRS score has been well accepted for long‐term outcome prediction and CRLM patient stratification,^1^ we were particularly interested in the effect of age on the prognosis of patients with different CRS scores. Two hundred forty‐seven patients, including 37 in the elderly group and 210 in the young group, had CRS scores of 1–2, while 235 patients, including 23 in the elderly group and 212 in the young group, had CRS scores of 3–5. Age was not associated with worse long‐term outcomes in patients with CRS scores of 1–2 (Figure S1). As shown in Table S2, the baseline characteristics of the patients with high CRS scores were all similar between the two groups. Post‐operative complications, including surgery‐related and general complications, hospital stay duration, operative time, intraoperative blood loss and transfusion were comparable between the two groups (Table S3). The Kaplan–Meier method and log‐rank test showed that age ≥ 70 years was associated with worse PFS (median, young group 8.60 months vs. elderly group 4.67 months, *p* = 0.042, Figure 2ssA) and worse OS (median, young group 42.0 months vs. elderly group 25.4 months, *p* = 0.011, Figure 2B). Univariate analysis identified age ≥ 70 years as a potential risk factor for PFS (*p* = 0.045, HR = 1.61, 95% CI 1.01–2.58). Subsequent multivariate analysis identified age ≥ 70 years (*p* = 0.046, HR = 1.62, 95% CI 1.01–2.62), R0 resection (*p* = 0.031, HR = 0.706, 95% CI 0.516–0.968) and major hepatic resection (*p* = 0.043, HR = 1.44, 95% CI 1.01–2.05) as independent predictors of PFS. Univariate analysis identified age ≥ 70 years as a potential risk factor for OS (*p* = 0.013, HR = 2.11, 95% CI 1.17–3.8). Subsequent multivariate analysis identified age ≥ 70 years (*p* = 0.008, HR = 2.34, 95% CI 1.26–4.35), neoadjuvant chemotherapy (*p* = 0.036, HR = 1.68, 95% CI 1.04–2.73), primary tumour T stage 3 or 4 (*p* = 0.032, HR = 3.62, 95% CI 1.12–11.72), adjuvant chemotherapy (*p* = 0.002, HR = 0.505, 95% CI 0.328–0.778) and major hepatic resection (*p* = 0.027, HR = 1.75, 95% CI 1.07–2.86) as independent predictors of OS (Table S4).

## 4 DISCUSSION

Whether advanced age is associated with worse outcomes for CRLM patients receiving hepatectomy is still under debate,^5^, ^9^, ^10^, ^11^, ^12^, ^13^, ^14^, ^15^, ^16^ and little is known about elderly patients receiving simultaneous resection of the primary tumour and liver metastases. Advanced age was generally associated with increased frailty, more comorbidities and poorer performance status.^4^ Hence, heterogeneity is usually present between different age groups, and few published articles adjusted for these differences with propensity score matching.^9^, ^29^ In this study, we evaluated the impact of age ≥ 70 years on the short‐term and long‐term outcomes of patients receiving simultaneous resection of CRLM and performed propensity score matching analyses to minimise selection bias.

Several previous studies reported increased post‐operative morbidities after hepatectomy for CRLM in elderly patients.^14^, ^30^, ^31^ In this study, a higher overall post‐operative complication rate was observed in the elderly group before (65% vs. 46.2%, *p* = 0.006) and after PSM1 (68.0% vs. 46.0%, *p* = 0.013). In the dataset after PSM1, the rates of both surgery‐related complications (44.0% vs. 32.2%, *p* = 0.167) and general complications (42.0% vs. 32.2%, *p* = 0.249) were higher in the elderly group, but neither reached statistical significance, suggesting that both categories of complications may be affected by advanced age. In addition, the elderly group patients developed more major complications and minor complications than the young group patients (*p* = 0.045).

Advanced age has been reported to be associated with other short‐term outcomes, including the length of hospital stay,^5^, ^9^, ^32^ operative time^13^, ^15^, ^33^ and intraoperative blood loss volume,^9^, ^32^ in CRLM patients receiving hepatectomy, while these associations were not observed in several other studies.^10^, ^11^, ^16^, ^22^ In this study, the length of hospital stay, operative time, intraoperative blood loss volume and blood transfusion rate were all similar between the young group and elderly group before and after PSM1. Differences in these short‐term outcomes reported in other studies of CRLM patients receiving hepatectomy were not observed in this study, which may be partially explained by this study population have been further selected for simultaneous resection. Therefore, the study population may be more homogenous compared to those in previous reports in which simultaneous resection and staged resection were both included.

In this study, age was confirmed as an independent risk factor in multivariate analysis (*p* = 0.004, HR = 2.57, 95% CI 1.37–4.82). Little is known about the effect of age ≥ 70 years on the survival of CRLM patients treated by simultaneous resection. In a systematic review and meta‐analysis conducted by Yin et al., age <70 years was recommended as one of the selection criteria for simultaneous resection.^21^ The only study reporting age ≥ 70 years as a risk factor for the survival of patients receiving simultaneous resection of CRLM in the systematic review and meta‐analysis of *Yin* et al. was the study of Tanaka et al. published in 2004, which retrospectively reviewed 39 patients with simultaneous resection and showed that age ≥ 70 years was associated with worse survival on univariate analysis but not on multivariate analysis.^22^ Age was not identified as an independent risk factor in the study of Tanaka et al. possibly because of their limited sample size and selection bias, as only elderly patients with more favourable disease characteristics tend to receive curative surgery, which may mask the detrimental effect of age on survival. The present study confirmed the results of previous studies with larger cohorts and minimised selection bias by propensity score matching. However, this result should be interpreted cautiously for the following reasons: (1) In this study, the cause of death information was missing for a large proportion of patients. Non‐cancer‐related deaths may have contributed to the worse OS of the elderly patients. Although the differences in baseline characteristics and short‐term outcomes were significantly reduced by PSM2, these differences were not eliminated. After PSM2, the elderly group still had a higher proportion of patients with comorbidities (55.3% vs. 48.3%, *p* = 0.437) than the young group. The higher rate of comorbidities, including hypertension, diabete and cardiac diseases, may have increased the incidence of non‐cancer‐related deaths in elderly patients. (2) Three hundred and thirty‐six patients (69.7%) in this study experienced disease recurrence. In these patients, the OS included survival after recurrence, which is largely affected by the site of recurrence, the tumour burden and the systematic and local treatments after the first progression of the disease.^34^, ^35^, ^36^ Hence, the OS results need to be validated by further prospective studies.

As OS was affected by the above‐mentioned factors, we consider that PFS is the more reliable measure of long‐term oncological outcomes in this study. No difference in PFS was found between the elderly group and the young group before and after PSM2, indicating that selected elderly patients with synchronous CRLM can achieve similar long‐term survival when received simultaneous resection. Based on these findings, we conclude that simultaneous resection for elderly CRLM patients is acceptable. Age ≥ 70 should not be considered as an absolute contraindication of simultaneous surgery. We believe whether to perform simultaneous resection for an elderly patient still requires a comprehensive evaluation of the individual's disease characteristics, comorbidities and performance status, and the final decision should be reached based on a consensus among a multidisciplinary team.^37^ Several established tools to predict the prognosis of CRLM patients may help with the decision‐making process, including the CRS score.^1^ In this study, subgroup analyses revealed that age ≥ 70 years was not associated with worse long‐term outcomes in patients with CRS scores of 1–2, while age ≥ 70 years was an independent risk factor for both PFS (*p* = 0.046, HR = 1.62, 95% CI 1.01–2.62) and OS (*p* = 0.008, HR = 2.34, 95% CI 1.26–4.35) in patients with CRS scores of 3–5. The results of the subgroup analysis confirmed that simultaneous resection of synchronous CRLM for elderly patients with CRS scores of 1–2 is acceptable, while further studies are required to determine the optimal management of elderly patients diagnosed with synchronous CRLM and have CRS scores of 3–5. Interestingly, for patients with CRS scores of 3–5, adjuvant chemotherapy was an independent protective factor for OS, while neoadjuvant chemotherapy was an independent risk factor for OS. Further studies are required to demonstrate the precise role of perioperative chemotherapy in CRLM patients with high CRS scores, which is beyond the scope of this article.

A strength of this study is that the differences in baseline characteristics between the young group and elderly group were balanced with propensity score matching. Additional propensity score matching was performed to adjust for differences in short‐term outcomes and analyse survival, as short‐term outcomes might affect CRLM patient survival.^27^, ^28^ The two propensity score matching analyses minimised the confounding effects of other variables and provided stronger evidence for the association between advanced age and the prognosis of CRLM patients receiving simultaneous resection.

This study also has several limitations. First, as simultaneous surgery was preferred for resectable synchronous CRLM at this institution, the number of elderly patients diagnosed with synchronous CRLM and treated with staged resection was too small to support reliable comparison. Further studies directly comparing the prognosis of elderly patients diagnosed with synchronous CRLM and treated by simultaneous surgery or staged surgeries with larger sample size are required to determine the optimal surgical approach for this population, especially for patients with CRS score of 3–5. Second, cause of death information was missing for a large proportion of the patients included in this study, rendering a cancer‐specific survival (CSS) analysis inappropriate. Further studies with CSS as an endpoint can distinguish cancer‐related and non‐cancer‐related deaths and better explain the differences in OS. Third, this is a single‐centre retrospective study. The results require further validation in other centres. Finally, the post‐operative complications in this study were recorded until hospital discharge, post‐operative complications may develop after hospital discharge. A multi‐centre prospective study may resolve the limitations of this study, verify our results and provide stronger evidence.

## 5 CONCLUSIONS

Although elderly patients treated by simultaneous resection of CRLM had increased risk of post‐operative complications compared to young patients, similar PFS can be achieved. Simultaneous resection for elderly CRLM patients is acceptable, and age ≥ 70 should not be considered as an absolute contraindication of simultaneous surgery. Further studies are required to determine the optimal treatment for elderly CRLM patients with high CRS score.

## FUNDING INFORMATION

This study was supported by the National Natural Science Foundation of China (81972311), the Non‐profit Central Research Institution Fund of Chinese Academy of Medical Sciences (2019PT310026) and Sanming Project of Medicine in Shenzhen (No. SZSM202011010). No funding source participated in either design of the study, data collection, analysis, interpretation or preparation of the manuscript.

## CONFLICT OF INTEREST

The authors declare that they have no competing interests.

## AUTHOR CONTRIBUTIONS

QC and HZ participated in the design of this study. QC, ZH, HZ and JC collected the data. QC, YZ and YD performed data analysis and interpretation. QC and YZ prepared the manuscript, which was further revised by all authors. All authors read and approved the final manuscript.

## ETHICS STATEMENT

This study was approved by the Institutional Review Board of the Cancer Hospital, Chinese Academy of Medical Sciences (ID:NCC2019C‐016) and conformed to the Declaration of Helsinki.

## Supporting information


Figure S1

Table S1‐S4

## Data Availability

The data that support the findings of this study are available upon request from the corresponding author. The data are not publicly available due to privacy or ethical restrictions.
