# Supplementary material for: Outcomes of simultaneous resection for elderly patients with colorectal liver metastasis: A propensity score matching analysis
Source: Cancer Med. 2022 May 24;11(24):4913–26. doi: 10.1002/cam4.4826 (PMC9761077; doi:10.1002/cam4.4826)

Supplementary Table 1 Detailed postoperative complications in unmatched full cohort

|  | Age<70 (n=422) | Age≥70 (n=60) | *P* |
| --- | --- | --- | --- |
| Complication (%) | 195 (46.2) | 39 (65.0) | 0.006 |
| Infection (%) | 77 (18.2) | 13 (21.7) | 0.525 |
| Anastomotic fistula (%) | 7 (1.7) | 0 (0) | 0.315 |
| haemorrhage (%) | 19 (4.5) | 8 (13.3) | 0.005 |
| ascites or hydrothorax (%) | 34 (8.1) | 4 (6.7) | 0.708 |
| Other complications (%) | 92 (21.8) | 17 (28.3) | 0.258 |

Supplementary Table 2 Demographics, clinicopathological features, surgery and chemotherapy details in patients with CRS score 3-5

|  | Age<70 (n=212) | Age≥70 (n=23) | *P* |
| --- | --- | --- | --- |
| Female (%) | 144 (67.9) | 17 (73.9) | 0.557 |
| BMI≥24 (%) | 101 (47.6) | 11 (47.8) | 0.987 |
| Comorbidity (%) | 83 (39.2) | 11 (47.8) | 0.42 |
| ASA score≥3 (%) | 17 (8.0) | 4 (17.4) | 0.135 |
| Primary site in colon (%) | 123 (58.0) | 16 (69.6) | 0.285 |
| Primary site in right hemicolon (%) | 36 (17.0) | 6 (26.1) | 0.279 |
| Bilobular distribution (%) | 130 (61.3) | 18 (78.3) | 0.11 |
| Number of liver metastases≥2 (%) | 196 (92.5) | 22 (95.7) | 0.574 |
| Diameter of largest liver lesion≥3 cm (%) | 115 (54.2) | 11 (47.8) | 0.558 |
| Poor differentiation (%) | 78 (36.8) | 10 (43.5) | 0.529 |
| Primary tumor T stage 3 or 4 (%) | 196 (92.5) | 21 (91.3) | 0.844 |
| Primary lymph node metastasis (%) | 208 (98.1) | 22 (95.7) | 0.437 |
| CEA≥10 ng/μl (%) | 98 (46.2) | 15 (65.2) | 0.083 |
| Extrahepatic metastasis (%) | 20 (9.4) | 3 (13.0) | 0.58 |
| Liver-first simultaneous resection (%) | 153 (72.2) | 17 (73.9) | 0.859 |
| Surgery procedure (%) |  |  | 0.867 |
| Totally laparoscopic | 34 (16.0) | 3 (13.0) |  |
| Mixed surgery | 123 (58.0) | 13 (56.5) |  |
| Totally open surgery | 55 (25.9) | 7 (30.4) |  |
| R0 resection (%) | 143 (67.5) | 15 (65.2) | 0.828 |
| Concomitant RFA (%) | 24 (11.3) | 3 (13.0) | 0.806 |
| Major hepatic resection (%) | 148 (69.8) | 19 (82.6) | 0.199 |
| Pringle manoeuvre (%) | 172 (81.1) | 20 (87.0) | 0.493 |
| Neoadjuvant chemotherapy (%) | 141 (66.5) | 12 (52.2) | 0.171 |
| Adjuvant chemotherapy (%) | 141 (66.5) | 16 (69.6) | 0.768 |

Supplementary Table 3 Short-term outcomes in patients with CRS 3-5

|  | Age<70 (n=212) | Age≥70 (n=23) | *P* |
| --- | --- | --- | --- |
| Postoperative hospital stay/d (median [IQR]) | 10.00 [9.00, 13.00] | 10.00 [9.00, 13.50] | 0.761 |
| Blood loss/ml (median [IQR]) | 200.00 [200.00, 400.00] | 200.00 [100.00, 600.00] | 0.932 |
| Operation time/min (median [IQR]) | 360.00 [290.00, 450.25] | 345.00 [285.00, 429.00] | 0.561 |
| Transfusion (%) | 49 (23.1) | 7 (30.4) | 0.434 |
| Complication (%) | 99 (46.7) | 15 (65.2) | 0.091 |
| Clavien-Dindo grade (%) |  |  | 0.455 |
| No complications | 113 (53.3) | 8 (34.8) |  |
| Minor (1-2) | 50 (23.6) | 10 (43.5) |  |
| Major (3-4) | 49 (23.1) | 5 (21.7) |  |
| Surgery-related complication (%) | 63 (29.7) | 6 (26.1) | 0.717 |
| General complications (%) | 68 (32.1) | 9 (39.1) | 0.494 |

Supplementary Table 4 Univariate and Multivariate analysis of factors associated with PFS and OS in patients with CRS score 3-5

|  | PFS | | | | OS | | | |
| --- | --- | --- | --- | --- | --- | --- | --- | --- |
|  | Univariate analysis | | Multivariate analysis | | Univariate analysis | | Multivariate analysis | |
| Factor | *P* | HR (95% CI) | *P* | HR (95% CI) | *P* | HR (95% CI) | *P* | HR (95% CI) |
| Age≥70 | 0.045 | 1.61 (1.01-2.58) | **0.046** | **1.62 (1.01-2.62)** | 0.013 | 2.11 (1.17-3.8) | **0.008** | **2.34 (1.26-4.35)** |
| Female | 0.955 | 1.01 (0.743-1.37) |  |  | 0.303 | 0.801 (0.525-1.22) |  |  |
| BMI≥24 | 0.722 | 1.05 (0.792-1.4) |  |  | 0.621 | 0.906 (0.613-1.34) |  |  |
| Comorbidity | 0.380 | 0.877 (0.654-1.18) |  |  | 0.583 | 0.895 (0.601-1.33) |  |  |
| ASA score≥3 | 0.146 | 1.42 (0.884-2.29) |  |  | 0.466 | 1.26 (0.675-2.36) |  |  |
| Primary site in colon | 0.421 | 0.889 (0.666-1.18) |  |  | 0.854 | 1.04 (0.697-1.54) |  |  |
| Primary site in right hemicolon | 0.082 | 0.7 (0.469-1.05) | 0.072 | 0.670 (0.433-1.036) | 0.746 | 1.09 (0.64-1.87) |  |  |
| Bilobular distribution | 0.146 | 1.25 (0.926-1.68) |  |  | 0.608 | 1.11 (0.741-1.67) |  |  |
| Number of liver metastases≥2 | 0.617 | 0.87 (0.505-1.5) |  |  | 0.809 | 1.11 (0.484-2.53) |  |  |
| Diameter of largest liver lesion≥3 cm | 0.049 | 1.33 (1-1.78) | 0.164 | 1.25 (0.914-1.71) | 0.006 | 1.77 (1.19-2.64) | 0.083 | 1.49 (0.950-2.34) |
| Poor differentiation | 0.804 | 0.963 (0.715-1.3) |  |  | 0.864 | 1.04 (0.681-1.58) |  |  |
| Primary tumor T stage 3 or 4 | 0.392 | 1.27 (0.736-2.19) |  |  | 0.015 | 4.18 (1.32-13.2) | **0.032** | **3.62 (1.12-11.72)** |
| Primary lymph node metastasis | 0.986 | 1.0 1(0.375-2.72) |  |  | 0.982 | 0.984 (0.242-4) |  |  |
| CEA≥10 ng/μl | 0.291 | 1.17 (0.877-1.55) |  |  | 0.049 | 1.49 (1-2.21) | 0.188 | 1.34 (0.866-2.08) |
| Extrahepatic metastasis | 0.052 | 1.59 (0.997-2.55) | 0.093 | 1.51 (0.934-2.45) | 0.766 | 0.896 (0.435-1.85) |  |  |
| Liver-first simultaneous resection | 0.249 | 1.21 (0.876-1.67) |  |  | 0.678 | 1.1 (0.706-1.71) |  |  |
| Surgery procedure |  |  |  |  |  |  |  |  |
| Totally laparoscopic |  | Ref |  |  |  | Ref |  |  |
| Mixed surgery | 0.142 | 1.38 (0.897-2.13) |  |  | 0.333 | 1.42 (0.701-2.86) | 0.450 | 0.750 (0.356-1.58) |
| Totally open surgery | 0.243 | 1.33 (0.823-2.15) |  |  | 0.082 | 1.927 (0.922-4.03) | 0.879 | 1.06 (0.490-2.31) |
| R0 resection | 0.004 | 0.639 (0.475-0.861) | **0.031** | **0.706 (0.516-0.968)** | 0.009 | 0.59 (0.397-0.875) | 0.285 | 0.790 (0.512-1.22) |
| Concomitant RFA | 0.080 | 1.47 (0.956-2.25) | 0.249 | 1.31 (0.826-2.091) | 0.005 | 2.02 (1.25-3.28) | 0.164 | 1.44 (0.862-2.41) |
| Major hepatic resection | <0.001 | 1.75 (1.26-2.43) | **0.043** | **1.44 (1.01-2.05)** | 0.007 | 1.92 (1.2-3.05) | **0.027** | **1.75 (1.07-2.86)** |
| Pringle manoeuvre | 0.232 | 1.26 (0.864-1.83) |  |  | 0.216 | 1.37 (0.831-2.27) |  |  |
| Neoadjuvant chemotherapy | 0.948 | 1.01 (0.749-1.36) |  |  | 0.083 | 1.47 (0.951-2.28) | **0.036** | **1.68 (1.04-2.73)** |
| Adjuvant chemotherapy | 0.738 | 1.05 (0.777-1.43) |  |  | 0.022 | 0.628 (0.421-0.935) | **0.002** | **0.505 (0.328-0.778)** |
| Blood loss≥200 ml | 0.708 | 1.07 (0.761-1.49) |  |  | 0.442 | 1.2 (0.758-1.89) |  |  |
| Operation time≥325 min | <0.001 | 1.72 (1.27-2.32) | 0.112 | 1.31 (0.940-1.83) | 0.002 | 1.96 (1.28-2.99) | 0.115 | 1.46 (0.912-2.35) |
| Transfusion | 0.892 | 0.977 (0.697-1.37) |  |  | 0.114 | 1.44 (0.917-2.25) |  |  |
| Postoperative hospital stay≥10 d | 0.188 | 1.22 (0.908-1.64) |  |  | 0.209 | 1.31 (0.861-1.98) |  |  |
| Clavien-Dindo grade |  |  |  |  |  |  |  |  |
| No complication |  | Ref |  |  |  | Ref |  |  |
| Minor | 0.053 | 1.40 (0.997-1.96) | 0.359 | 1.18 (0.827-1.69) | 0.827 | 1.08 (0.560-2.07) |  |  |
| Major | 0.141 | 1.31 (0.915-1.88) | 0.813 | 1.05 (0.719-1.52) | 0.864 | 1.07 (0.493-2.32) |  |  |
| Surgery-related complication | 0.145 | 1.26 (0.923-1.72) |  |  | 0.236 | 1.29 (0.846-1.97) |  |  |
| General complications | 0.418 | 1.13 (0.838-1.53) |  |  | 0.524 | 1.14 (0.758-1.72) |  |  |

Supplementary Figure. Survival plot comparing survival in subgroup analyses of patients with CRS score 1-2. (a) PFS (b) OS.


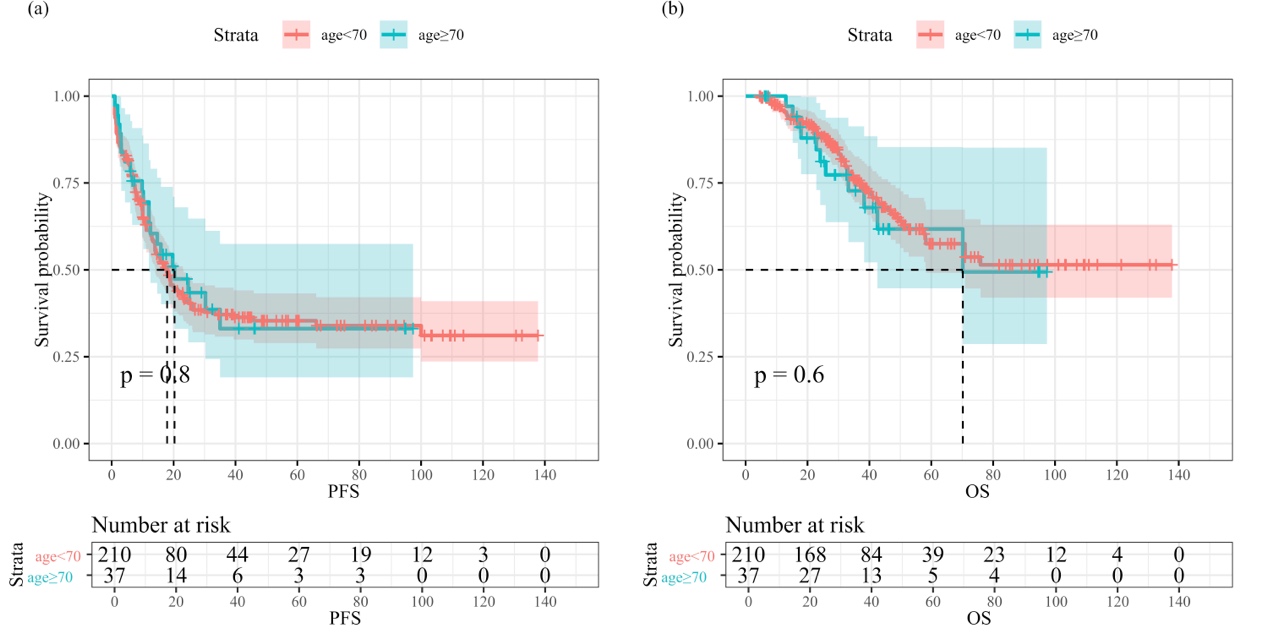

Supplement: Supplementary file 2 — Table S1‐S4 [file CAM4-11-4913-s002.docx]
